# Frequency and determinants for hemorrhagic transformation of posterior cerebral stroke: Posterior ischemic stroke and hemorrhagic transformation

**DOI:** 10.1186/s13104-017-2889-x

**Published:** 2017-11-13

**Authors:** Francesca Valentino, Luana Gentile, Valeria Terruso, Sergio Mastrilli, Paolo Aridon, Paolo Ragonese, Caterina Sarno, Giovanni Savettieri, Marco D’Amelio

**Affiliations:** 1 Dipartimento di Biomedicina Sperimentale e Neuroscienze Cliniche (BioNeC), Università degli Studi di Palermo, Via Gaetano La Loggia 1, 90129 Palermo, Italy; 2 Dipartimento di Neurologia e Psichiatria, Università degli Studi di Roma “La Sapienza”, Rome, Italy; 3 ARNAS Civico, Di Cristina e Benfratelli, Palermo, Italy; 4 Azienda Ospedaliera Universitaria Policlinico P. Giaccone, Palermo, Italy

**Keywords:** Posterior ischemic stroke, Hemorrhagic transformation, Epidemiology

## Abstract

**Background:**

hemorrhagic transformation is a threatening ischemic stroke complication. Frequency of hemorrhagic transformation differs greatly among studies, and its risk factors have been usually studied in patients with anterior ischemic stroke who received thrombolytic therapy. We evaluated, in a hospital-based series of patients with posterior ischemic stroke not treated with thrombolysis, frequency and risk factors of hemorrhagic transformation. Patients with posterior circulation stroke were seen in our Department during the period January 2004 to December 2009. Demographic and clinical information were collected. We estimated risk for spontaneous hemorrhagic transformation by means of uni- and multivariate logistic regression analyses.

**Results:**

119 consecutive patients were included (73 males, 61.3%). Hemorrhagic transformation was observed in 7 patients (5.9%). Only clinical worsening was significantly associated with hemorrhagic transformation (OR 6.8, 95% CI 1.3–34.5).

**Conclusions:**

Our findings indicate that patients with posterior have a low risk of spontaneous hemorrhagic transformation, suggesting that these patients might have greater advantage from intravenous thrombolysis.

## Background

Neurological and medical complications of ischemic stroke (IS) are responsible for higher morbidity and mortality. The former occur earlier than medical complication, and they affect outcomes with potential serious short- and long-term consequences [1]. Though, thrombolytic therapy significantly reduces the proportion of patients dead or dependent in activities of daily living, it also significantly increases the risk of hemorrhagic transformation (HT) of the ischemic lesion, complication that independently from more aggressive treatment is associated with an overall increased mortality [2].

We have previously investigated frequency and risk factors for HT of the anterior ischemic stroke [3, 4].

Compared to anterior, posterior stroke though less frequent, covering one-fifth of all IS [5] is characterized by higher morbidity and mortality [6]. Frequency and risk factors for spontaneous HT of the posterior ischemic stroke received less attention than those of the carotid artery territory.

We have investigated frequency and risk factors for spontaneous HT in a sample of patients with posterior IS, treated only with conventional therapy, not undergoing thrombolysis. To individuate patients with posterior ischemic stroke suitable for thrombolytic therapy, it is critical to estimate frequency of spontaneous HT among ischemic stroke patients, and determine its risk factors.

## Methods

All consecutive patients diagnosed with posterior circulation stroke (PCS) in our Department during the period January 2004 to December 2009, were enrolled in this study. Stroke was defined as “rapidly developing clinical symptoms and/or signs of focal, and at times global loss of cerebral function, with symptoms lasting 24 h or leading to death, with no apparent cause other than that of vascular origin” [7]. Only patients who performed a baseline brain computed tomography (CT) scan within 24 h from symptoms onset and a follow-up CT within 7 days from symptoms onset were included in this study.

Patients with transient ischemic attacks (TIA) and cerebral hemorrhage were excluded.

Hemorrhagic transformation was defined as ‘any degree of hyperdensity within the area of low attenuation’ [8] and graded using European Cooperative Acute Stroke Study (ECASS) classification [9]. ECASS classifies hemorrhagic transformation into two classes: hemorrhagic infarction (HI) and parenchymal hematoma (PH). Each class is further divided into two subtypes. For HI: HI-1 is defined as small petechiae along the margins of the infarcted area, while HI-2 is characterized by confluent petechiae within the infarcted area, in the absence of any mass effect. For PH: PH-1 is defined as hematoma in less than 30% of the infarcted area in the presence of only mild mass effect, while PH-2 as hematoma in more than 30% of the parenchymal lesion with an evident mass effect.

We investigated the following risk factors along with demographic variables: history TIA, ischemic or hemorrhagic stroke, myocardial infarction, atrial fibrillation, diabetes, or arterial hypertension. Cardioembolic origin of stroke was classified according to the Trial of Org 10,172 in Acute Stroke Treatment criteria [10]. Continuous variables as determined at patient admission (values of systolic and diastolic blood pressure, glycemia, platelet count, international normalized ratio, total cholesterol) were dichotomized according to their median value. History of smoking and alcohol consumption were categorized in ever smokers versus non-smokers, and ever drinker versus non-drinker.

At a first CT scan, the presence of early signs of IS, focal hypodensity consistent with the clinical symptoms, swelling due to developing infarction, blurring of grey matter-white matter distinction, were all assessed by a neuroradiologist (C.S.) who was blinded to the clinical characteristics of stroke and its progress towards HT.

Level of consciousness at admission was categorized in normal/mild (patient alert and responsive to normal or loud verbal stimuli) to moderate/severe impairment (responsive only to painful stimuli or unresponsive). Clinical worsening was defined as worsening of the motor deficit and/or the level of consciousness. Written informed consent to participate in the study was obtained from patients. When patients could not give the consent themselves, written assent from the next of kin was obtained. The study, as well as informed consent signed by the patient or by the next of kin, were approved by the local ethic committee of the Palermo University Hospital “Paolo Giaccone”.

### Statistical analysis

Clinical characteristics of patients with and without HT were compared using Chi square test for categorical variables and t test for continuous variables.

As a measure of association between HT (dependent variable) and the variables investigated (independent variables) we calculated odds ratios (OR) and 95% confidence intervals (CI), by means of logistic regression analysis. These were categorized as it has been described in the section methods. The association was considered statistically significant for p < 0.05.

If at univariate analysis, more that a variable was found significantly associated with HT, a model including these variables would have been built (multivariate analysis). Statistical analysis was performed using SAS Statistical Package Version 9.1.

## Results

One-hundred fifty-six patients were admitted to our Department during the study period for symptoms attributable to posterior circulation. Thirty-seven patients, who underwent the first brain CT more than 24 h from symptoms onset or patients with cerebral hemorrhage or TIA, were not included in the study. Finally, 119 patients were included (73 males, 61.3%). HT was found in 7 patients (5.9%). Parenchymal hematoma (PH-1 in 2, PH-2 in 1) occurred in 3 patients while petechial hemorrhages were observed in 4 (HI-1 in 4). Difference of median age at admission between patients with (74.5 ± 8.6) and without HT (70.3 ± 11.3) was not significant (p = 0.3). Table 1 shows the association between HT and some demographic data, clinical parameters, neurological status on admission, radiological findings and risk factors for stroke. Cardioembolic origin of stroke was more frequent in patients with HT (12.9%) compared to those without HT (3.4%) (p = 0.07). Only clinical worsening was still significantly associated with HT (Table 1).

**Table 1:** Demographic and clinical characteristics of patients with and without HT

	HT/all IS (%)	OR (5% CI)	*p*
Gender			
Female	3/46 (6.5)		
Male	4/73 (5.5)	0.8 (0.2, 3.9)	0.8
Age			
< 72.4	2/60 (3.3)		
≥ 72.4	5/59 (8.5)	2.7 (0.5, 14.4)	0.2
Consciousness^a^			
Normal/mild impairment	6/100 (6.0)		
Moderate/severe impairment	1/18 (5.6)	0.9 (0.1, 8.1)	0.9
Clinical worsening^a^			
No	4/104 (3.8)		
Yes	3/14 (21.4)	6.8 (1.3, 34.5)	0.02
Early CT signs			
No	4/95 (4.2)		
Yes	3/24 (12.5)	3.2 (0.7, 15.6)	0.1
Infarct size			
Small	0/68 (0)		
Medium to large	7/51 (13.7)	–	na
Cardioembolic source			
No	3/88 (3.4)		
Yes	4/31 (12.9)	4.2 (0.9, 19.9)	0.07
Atrial fibrillation			
No	4/95 (4.2)		
Yes	3/24 (12.5)	3.2 (0.7, 15.6)	0.1
Hypertension^a^			
No	0/31 (0)		
Yes	7/85 (8.2)	–	na
Diabetes^a^			
No	3/78 (3.9)		
Yes	4/39 (10.3)	2.9 (0.6, 13.5)	0.2
Dyslipidemia^a^			
No	4/89 (4.5)		
Yes	3/26 (11.5)	2.8 (0.6, 13.3)	0.2
Previous CHD^a^			
No	4/87 (4.6)		
Yes	3/27 (11.1)	2.6 (0.5, 12.4)	0.2
Smoking habit			
No	3/61 (4.9)		
Yes	4/58 (6.9)	1.4 (0.3, 6.7)	0.6
Alcohol			
No	4/49 (8.2)		
Yes	3/70 (4.3)	0.5 (0.1, 2.4)	0.4

*CHD* coronary hearth disease

^a^Data may be not available for all patients

No significant differences were shown for the other variables investigated (values of systolic and diastolic blood pressure, platelet count, glycemia, international normalized ratio, total, HDL and LDL cholesterol, and triglycerides) (Table 2).

**Table 2:** Clinical parameters of patients with and without HT

	HT (%)	OR (5% CI)	*p*
SBP, mmHg^a^			
< 140	3/67 (4.5)		
≥ 140	4/48 (8.3)	1.9 (0.4–9)	0.4
DBP, mmHg^a^			
< 80	5/74 (6.8)		
≥ 80	2/41 (4.9)	0.7 (0.1–3.8)	0.7
Platelets, 10^3^/µl^a^			
< 214	3/57 (5.3)		
≥ 214	4/57 (7.0)	0.7 (0.15–3.4)	0.6
INR*			
< 1	2/36 (5.6)		
≥ 1	5/74 (6.8)	1.2 (0.2–6.7)	0.6
Total cholesterol, mg/dl^a^			
< 193	3/51 (5.9)		
≥ 193	4/53 (7.5)	1.3 (0.3–6.1)	0.7
HDL, mg/dl^a^			
< 45.5	4/63 (6.3)		
≥ 45.5	3/37 (8.1)	0.8 (0.1–3.6)	0.7
LDL, mg/dl^a^			
< 109.4	4/40 (10.0)		
≥ 109.4	2/49 (4.0)	0.4 (0.06–2.2)	0.3
Triglycerides, mg/dl^a^			
< 113.5	3/38 (7.9)		
≥ 113.5	4/66 (6.0)	0.7 (0.1–3.5)	0.7
Blood glucose, mg/dl^a^			
< 125.5	3/74 (4.0)		
≥ 125.5	4/38 (10.5)	2.8 (0.6–13.1)	0.2

*SBP* systolic blood pressure, *DBP* diastolic blood pressure

^a^Data may be not available for all patients

## Discussion

Main finding of our study is a low incidence of spontaneous HT in patients with posterior ischemic stroke (5.9%) that reaches even lowest values if we consider only PH (2.5%). HT was significantly associated with clinical worsening.

To our knowledge, this is the first study investigating risk of spontaneous HT in patients with PCS. Indeed, most authors investigating hemorrhagic transformation have focused their attention on anterior circulation stroke (ACS), either in patients treated or not with recanalization therapy [11–13].

Measure of HT risk in patients with PCS [14] derives from a small sample study investigating frequency and relationship of HT to disruption of blood–brain-barrier, detected by MRI. Hemorrhagic transformation was observed in 5 (35.7%) out of the 14 patients included. In this clinical study, however HT was observed only in patients who received pharmacological thrombolysis combined with mechanical rechanalization. Therefore, the observed reduced frequency of HT in our study (6%) when compared to Lee study (35%) very likely depends on the more aggressive treatment of the latter. The overall lower incidence compared to ACS [15] might find an alternative explanation considering stroke territory involvement.

One of the major strength of our study is the possibility to compare frequency of HT between anterior and posterior IS in a population sample enrolled in the same study period using the same methods. In a larger sample of patients with anterior ischemic stroke, we found a frequency of spontaneous HT of 12% [3]. As study period of enrollment of the patients, inclusion and exclusion criteria, and clinical assessment were the same of current study, difference of frequency of HT between anterior and posterior stroke circulation is very likely attributable to stroke territory. No patients in both groups underwent thrombolysis and no difference existed for treatment between the two populations (anterior and posterior stroke). It is possible that smaller size of ischemic area of PCS might be the explanation of a reduced incidence of HT in posterior compared to anterior stroke.

In a study enrolling all patients undergoing intravenous thrombolysis, no symptomatic intracranial hemorrhage was observed in posterior as compared to a 5% in the anterior stroke circulation [15]. The same authors attributed to an hypothetical reduced volume of infarcts of the PCS as compared to larger volume of the ACS.

Lower risk of HT, in PCS as compared to ACS, might depend on the fact that occlusions of posterior circulation arteries typically cause ischemic lesion of smaller sizes, which may reduce the hemorrhagic transformation rate. As suggested [16], smaller vessel calibers of posterior circulation, especially those of brain stem that is nourished by small end-arteries, might be the explanation of smaller size infarcts in PCS. In addition, collaterals of the posterior circulation might “functionally work” better than those of the anterior circulation [17].

Because the size of ischemic stroke [3, 18] was an independent risk factor for HT in ACS stroke, higher frequency of smaller lesion volume in infratentorial compared to supratentorial strokes [19] might partially explain lower incidence of hemorrhagic transformation in patients with posterior ischemic stroke.

It is well known that size of HT has a great influence on the prognosis. All HT observed in our patients with clinical worsening were PH (2 PH1, 1 PH2). Fiorelli [20] reported for both the placebo and the treatment groups an increased risk of early neurological deterioration and of 3-month death only when PH size was greater than 30% of the infarcted area. We have also later demonstrated [2] that only patients with spontaneous PH and not those with HI had a significant eight fold increased risk of death at 30 and 90 days from stroke onset. As suggested, to establish whether neurological worsening is due to HT itself or due to the size of infarct and the associated edema concurrent with HT is still fundamental in thrombolysis or other stroke trials [21].

Although only clinical worsening seems to be the main predictor of HT in patients with PCS “has been modified. The new sentence is: “Although HT in patients with PCS is significantly associated only with clinical worsening, very likely preceding it, other variables such as cardioembolism, systemic hypertension, dyslipidemia are in our study associated with HT, and lack of significance of their association is very likely attributable to the small number of overall HT in our sample.

Cardioembolism is frequently reported as risk factor for HT [22], usually causing a large vessel occlusion and determining larger infarct and more severe stroke [23]. This data suggest that infarct size could reflect the embolic origin that could therefore represents the final cause of HT. However, atrial fibrillation and infarct area independently correlated with risk of spontaneous HT in patients with ACS [3, 18]. Atrial fibrillation is associated with greater infarct size, more frequent severe HT, and worst stroke outcomes [24].

Conversely, in a large US hospital registry study of 407 patients with PCS, embolism was the most common mechanism (40% of patients) [25] while large artery occlusive lesions was the second cause of hemodynamic brain ischemia (32% of patients). Infarcts most often included the distal posterior circulation territory, with the proximal and the middle territories were equally involved.

Main limits of our study, due to methodological issue, are the lack of clinical data (NIHSS scores) and of the absence of routine MRI scan as neuroimage exam. Regarding NIHSS is important to underline that it has already been demonstrated that it has limitations in detecting neurologic deficits of posterior circulation [26], though MRI has advantages for the study of the posterior circulation stroke, CT scan remains the exam more frequently used in the emergency setting.

Summing up, our data indicate that patients with PCS compared to those with ACS have a lower risk of spontaneous hemorrhagic transformation, suggesting that these patients might have a greater net benefit from intravenous thrombolysis.

## Conclusions

Results of our study indicate that patients with posterior compared to those with anterior stroke seem to have a lower risk of spontaneous hemorrhagic transformation, finding that might depend by smaller ischemic lesion volumes characterizing posterior stroke. When HT occurs in patients with PS, this is significantly associated with worsening of symptoms.

## Abbreviations

IS: ischemic stroke
HT: hemorrhagic transformation
PCS: posterior circulation stroke
ACS: anterior circulation stroke
CT: computed tomography
TIA: transient ischemic attacks
ECASS: European Cooperative Acute Stroke Study
HI: hemorrhagic infarction
PH: parenchymal hematoma
